# Intra-abdominal infection (IAI) following cesarean section: a retrospective study in a tertiary referral hospital in Egypt

**DOI:** 10.1186/s12884-019-2394-4

**Published:** 2019-07-08

**Authors:** Ahmed R. Abdelraheim, Khaled Gomaa, Emad M. Ibrahim, Mo’men M. Mohammed, Eissa M. Khalifa, Ayman M. Youssef, Ahmed K. Abdelhakeem, Heba Hassan, Ahmed Abd Alghany, Saad El Gelany

**Affiliations:** Department of Obstetrics &Gynecology, Minia Maternity & Children University Hospital, Faculty of Medicine, Minia University, Minia, Egypt

**Keywords:** Intra-abdominal infection, Cesarean section, Risk factors, Predictors

## Abstract

**Background:**

The incidence of post cesarean intra-abdominal infection (IAI) and the independent risk factors associated with it were retrospectively studied at a tertiary referral hospital in Egypt.

**Methods:**

The study targeted the period between January 2014 and December 2017 (4 years) at Minia University Hospital for Obstetrics and Gynecology (a tertiary referral hospital), Minia Governorate, Egypt. All cases that developed IAI following cesarean section (CS) during the study period were included (408 cases, which served as the case group); in addition, 1300 cases that underwent CS during the study period and were not complicated by IAI or surgical site Infection (SSI) were randomly chosen from the records (control group). The records of cases and controls were compared and bivariate analysis and multivariate logistic regression were used to identify risk factors for IAI.

**Results:**

During the studied period, there were 35,500 deliveries in the hospital, and 14200 cases (40%) of these were by cesarean section, producing a rate of 40%. The incidence of IAI post CS was 2.87%, and the mortality rate was 1.2% (due to septicemia). The most identifiable risk factors for IAI were chorioamnionitis (AOR 9.54; 95% CI =6.15–16.2; *p* ≤ 0.001) and premature rupture of membranes (PROM) (AOR 7.54; 95% CI =5.69–10.24; p ≤ 0.001). Risk factors also included: prolonged duration of CS >  1 h (AOR 3.42; 95% CI =2.45–5.23; *p* = 0.005), no antenatal care (ANC) visits (AOR 3.14; 95% CI =2.14–4.26; *p* = 0.003), blood loss > 1000 ml (AOR 2.86; 95% CI =2.04–3.92; *p* = 0.011), emergency CS (AOR 2.24; 95% CI =1.78–3.29; *p* = 0.016), prolonged labor ≥24 h. (AOR 1.76; 95% CI =1.26–2.27; *p* = 0.034) and diabetes mellitus (AOR 1.68; 95% CI =1.11–2.39; *p* = 0.021).

**Conclusions:**

The incidence of IAI post CS in our hospital was 2.87%. Identification of predictors and risk factors for IAI is an important preventive measure.

## Background

Cesarean section (CS) is a surgical procedure for delivery of the fetus through incisions in the abdominal and the uterine walls, and it is the most common major operation performed in obstetrics [1]. Globally, the prevalence of CS is approximately 18.6%, ranging from 6 to 27.2%*.* The average rate of CS in North Africa is 27.8% [2], with high rate in Egypt (51.8%) [3]. Cesarean section is considered the most important risk factor for postpartum maternal infection, with women undergoing CS having a 5- to 20-fold higher chance of infection compared to those who delivered vaginally [4]. Infections can be in the organs, within the pelvis, or around the surgical site; these sometimes dangerous infections can increase maternal morbidity, lengthen hospital stays, and increase medical costs and can increase maternal mortality by a considerable amount (32.5%) [5]. It has been reported that the rate of wound infection post CS ranges from 3 to 16% [6].

Generally, IAIs are common surgical emergencies and have been recognized as major contributors to mortality in emergency departments globally, “particularly if not well managed” [7]. Recently, it has been reported that the rate of IAI following CS was 3% in one study [8], and it was 4.9% in another study [9]*.* Knowledge of the predictors and risk factors associated with IAI is pivotal in reducing the risk of infection and helping to develop prevention strategies. Several factors were reported as risk factors for post cesarean infection, including duration of labor prior to the CS, premature rupture of membranes (PROM), chorioamnionitis, subcutaneous hematoma, limited antenatal care (ANC) visits, previous and emergency CS, prolonged operative time, excessive blood loss, and postoperative anemia [6]. However, diabetes mellitus (DM), obesity, surgeon experience and maternal age (> 35 years) were implicated by others [10]. The objectives of this study were to identify the rate of IAI after CS and to determine risk factors predictive for it.

## Methods

### Study settings

This is a retrospective case control study which targeted the period between January 2014 and December 2017 at Minia University Hospital for Obstetrics and Gynecology, Minia Governorate, Egypt. This hospital is the only tertiary hospital in Minia Governorate, receiving referrals from nine district maternity units and serving a population of 5.5 million.

### Study population

During the study period, there were a total of 35500 deliveries in the hospital. Of these, 14200 deliveries were by CSs. A total of 408 cases developed IAI post CS during the study period; these were considered to be the case group (IAI patients). A control group of 1300 cases that underwent CS during the study period without developing IAI or SSI were randomly chosen from the records (a simple randomization method was used). Patients with IAI were identified using the definitions from the Centers for Disease Control and Prevention’s National Nosocomial Infections Surveillance System [11, 12].

### Data collection

The data were obtained from records of the labor ward birth registers by well-trained persons. All patients who developed intra-abdominal infection post CS during the studied period were included with no exclusions; however, for the control group, cases were excluded if their records had excessive missing data. Demographic data for each patient, including age; parity; body mass index (BMI) on admission; previous obstetric history including previous CS; antenatal care; and medical complications during pregnancy, such as DM, gestational age at delivery, PROM, duration of labor, operative time, blood loss during CS, and use of antibiotics, were noted from each patient’s medical, delivery and operating room records.

### Ethical considerations

The research project was approved by the ethical committee of the Department of Obstetrics and Gynecology, Minia University Hospital (reference number: MOBGYN: 0040).

### Statistical analyses

Statistical analyses were performed by using SPSS (version 21.0). Categorical variables were compared by using Chi-square or Fisher exact test; however, continuous variables were presented as the mean ± SD and were compared using the independent sample T test. Univariate and multivariate logistic regression analyses were conducted for determinations of risk factors associated with IAI. Adjusted odds ratios (AORs) were estimated from the logistic models with 95% confidence intervals (CIs). A probability value of 0.05 was considered as significant, and P. value of 0.01 was considered as highly significant.

## Results

During the studied period, the total number of deliveries in the hospital was 35500 cases, of which 14200 cases (40%) were delivered by CS. Of these CS deliveries, 408 cases developed IAI, producing an incidence of 2.87%.

Table 1 presents the baseline demographics and obstetric variables for IAI patients and the control group. There were no significant differences between groups regarding residence, parity and gestational age; however, women who developed IAI had a significantly higher mean age compared to controls (*P* = 0.006). Women who developed IAI had a higher incidence of emergency CS compared to those who did not (83.4% vs. 64.8%; *p* < 0.001, COR = 2.71); also, the number of cases who had operative time “duration of CS” (> 1 h) was significantly higher in IAI group (*P* ≤ 0.001, COR = 3.95). We noticed that cases with blood loss > 1000 ml were significantly higher in IAI group compared to control (12.5% vs. 4.3%, *p* ≤ 0.001, COR = 3.17); additionally, the same trend was observed in cases with prolonged duration of labor ≥24 h. Furthermore, we found that IAI post CS was significantly associated with high incidence of chorioamnionitis (20.1%, P. ≤0.001, COR = 11.02), PROM (57.6%, P. ≤0.001, COR = 8.32), no ANC visits (43.6%, P. ≤0.001, COR = 3.49) and DM (8.6%, P. ≤0.011, COR = 1.72), while obesity was not associated significantly with IAI.

**Table 1:** Baseline and obstetric data in cases and control groups

Variable	Cases group (*n* = 408)	Control group (*n* = 1300)	*X* ^*2*^	*P* value (Sig.)	Crude Odds ratio (95% CI)
Age (mean ± SD)	31.5 ± 5.2	30.7 ± 4.6	2.75^#^	**0.006**	–
< 19	39 (9.6%)	113 (8.7%)	4.90	0.09	1.15 (0.78–1.69)
20–34	326 (79.9%)	1092 (84.0%)			1.0
≥ 35	43 (10.5%)	95 (7.3%)			1.52 (1.03–2.21)
Residence					
Urban	171 (41.9%)	577 (44.4%)	0.77	0.38	1.0
Rural	237 (58.1%)	723 (55.6%)			1.11 (0.88–1.38)
Parity					
1–4	364 (89.2%)	1184 (91.1%)	1.27	0.25	1.0
> 4	44 (10.8%)	116 (8.9%)			1.23 (0.85–1.77)
Gestational age					
< 37 wks.	54 (13.2%)	147 (11.3%)	1.11	0.29	1.20 (0.86–1.67)
≥ 37 wks.	354 (86.8%)	1153 (88.7%)			1.0
Type of the CS					
Elective	68 (16.6%)	458 (35.2%)	50.2	≤ 0.001**	1.0
Emergency	340 (83.4%)	842 (64.8%)			2.71 (2.04–3.61)
Duration of CS					
≤ 1 h	39 (9.5%)	383 (29.5%)	66.1	≤ 0.001**	1.0
> 1 h	369 (91.5%)	917 (70.5%)			3.95 (2.78–5.61)
Blood loss (> 1000 ml)					
No	357 (87.5%)	1244 (95.7%)	35.5	≤ 0.001**	1.0
Yes	51 (12.5%)	56 (4.3%)			3.17 (2.13–4.72)
Duration of labor					
< 24 h	359 (88.0%)	1219 (93.8%)	14.8	≤ 0.001**	1.0
≥ 24 h	49 (12.0%)	81 (6.2%)			2.05 (1.41–2.98)
Chorioamnionitis					
No	326 (79.9%)	1271 (97.8%)	163.1	≤ 0.001**	1.0
Yes	82 (20.1%)	29 (2.2%)			11.02 (7.09–17.1)
PROM					
No	173 (42.4%)	1116 (85.9%)	316.1	≤ 0.001**	1.0
Yes	235 (57.6%)	184 (14.1%)			8.32 (6.41–10.58)
ANC visits					
No	178 (43.6%)	276 (21.2%)	84.1	≤ 0.001**	3.49 (2.60–4.70)
1–4	143 (35.1%)	552 (42.5%)		≤ 0.001**	2.48 (1.91–3.23)
> 4	87 (21.3%)	472 (36.3%)			1.0
Obesity					
No	363 (89.0%)	1173 (90.2%)	0.54	0.46	1.0
Yes	45 (11.0%)	127 (9.8%)			1.14 (0.80–1.64)
Diabetes Mellitus					
No	373 (91.4%)	1233 (94.8%)	6.45	0.011*	1.0
Yes	35 (8.6%)	67 (5.2%)			1.72 (1.13–2.64)

# *P* value * Significant (*P* ≤ 0.05). ** Highly significant (*P* ≤ 0.01)

Table 2 shows management of IAI patients, the majority of cases (355 cases, 87.0%) were managed by re-exploration and drainage of pus; however, 34 cases (8.3%) were managed conservatively. We noticed that the rate of IAI increases with the increase of the number of scrubbed personnel (this means that despite sterilization, infection increases with multiple hands). In about one third of IAI cases, the CS scar was not open, and pus collected in the peritoneum. Regarding the type of suture used during the CS, Vicryl was used in 216 cases (52.9%) and chromic cat gut was used in 94 cases; the rest of cases were treated by unknown type of suture. Five cases from the total IAI cases (408) died due to septicemia with a rate of 1.22%.

**Table 2:** Management of IAI patients

Variable		Descriptive Cases group (*n* = 408) No. (%)
Decision	Re-explore and drainage of pus	355 (87.0%)
	Managed conservatively	34 (8.3%)
	Skin only was opened	19 (4.7%)
Scrubbed personnel	1 Surgeon and 1 nurse	44 (10.8%)
	1 Surgeon, 1 nurse and 1 assistant	62 (15.2%)
	1 Surgeon, 1 nurse and 2 assistants	88 (21.5%)
	1 Surgeon, 2 nurses and 2 assistants	99 (24.3%)
	2 surgeons, 1 nurse and 3 assistants	115 (28.2%)
Intra-operative findings during re-exploration	Scar of CS was open and pus collected in pelvis	119 (29.1%)
	Scar of CS was open and pus collected in sub-hepatic region	91 (22.3%)
	Scar of CS was open and pus collected in all abdomen	40 (9.8%)
	Scar of CS not open and pus collected in peritoneum	153 (37.5%)
	With missed towels	5 (1.3%)
Type of suture used during the CS	Vicryl	216 (52.9%)
	Chromic gut	94 (23.0%)
	Unknown suture	98 (24.1%)

Table 3 demonstrates the results of the multivariate analysis of risk factors for intra-abdominal infection post CS. The results revealed that age, parity, gestational age and obesity were not associated significantly with the rate of IAI and could not be considered as risk factors. However, risk factors for IAI were emergency CS (AOR 2.24; 95% CI =1.78–3.29; *p* = 0.016), duration of CS >  1 h (AOR 3.42; 95% CI =2.45–5.23; *p* = 0.005), blood loss > 1000 ml (AOR 2.86; 95% CI =2.04–3.92; *p* = 0.011), prolonged labor ≥24 h. (AOR 1.76; 95% CI =1.26–2.27; *p* = 0.034), no ANC visits (AOR 3.14; 95% CI =2.14–4.26; *p* = 0.003) and pregnancies complicated by diabetes mellitus (AOR 1.68; 95% CI =1.11–2.39; *p* = 0.021). Additionally, the results illustrated that the most identifiable risk factors were chorioamnionitis (AOR 9.54; 95% CI =6.15–16.2; *p* ≤ 0.001) and PROM (AOR 7.54; 95% CI =5.69–10.24; *p* ≤ 0.001).

**Table 3:** Multivariate analysis of risk factors for intra-abdominal infection

Variables	Adjusted Odds ratio (AOR) (95% CI)	*P* value (Sig.)
Age (≥ 35 years)	0.89 (0.68–1.67)	0.192^NS^
Parity (> 4)	0.86 (0.72–1.78)	0.413^NS^
Gestational age (< 37 wks.)	0.52 (0.66–1.42)	0.522^NS^
Type of the CS (Emergency)	2.24 (1.78–3.29)	0.016*
Duration of CS (> 1 h)	3.42 (2.45–5.23)	0.005**
Blood loss (> 1000 ml)	2.86 (2.04–3.92)	0.011*
Duration of labor (≥24 h.)	1.76 (1.26–2.27)	0.034*
Chorioamnionitis	9.54 (6.15–16.2)	≤ 0.001**
PROM	7.54 (5.69–10.24)	≤ 0.001**
No ANC visits	3.14 (2.14–4.26)	0.003**
Obesity	0.75 (0.61–1.47)	0.631
Diabetes Mellitus	1.68 (1.11–2.39)	0.021*

*NS* = Not significant

## Discussion

Intra-abdominal infection is one of the major causes of morbidity and mortality, particularly if poorly managed. The incidence of IAI post CS reported in the literature was 3% [8] and 4.9% [9], and in our study, the incidence was 2.87%, which is comparable with these findings. Very few data are available about the incidence of IAI post CS: most literature studied the incidence SSI following CS, such as an Ethiopian study, in which the incidence of SSI among women who had CS was 11.4% [13]. Additionally, studies from Cameroon [14], Nigeria [10] and Tanzania [15] reported a prevalence of 9–13%. In Egypt, some studies were conducted to provide evidence concerning the magnitude of healthcare-associated infections in different health care settings. In Kasr El-Aini University Hospitals (Egypt), it was found that the overall SSI rate was 12.1% [16]: the case fatality rate was 2.2% among admitted patients, and wound infections constituted 91.3% of all nosocomial infections, while another study reported a rate of 5.1% for postoperative nosocomial infection [17].

No significant differences were found between the case and control groups regarding residence and parity, which agreed with other studies [10]*.* In the present study, the case group had a higher incidence of emergency CS compared to the control group. In the case group, there was a higher number of patients with operative time > 1 h. In general, many authors reported that patients undergoing emergency CS are at higher risk of infection [9, 18–20]; this increased risk may be due to inadequate preparation time owing to maternal or fetal threat. Similar findings were reported by Killian et al. [21]. Also, it has also been reported that prolonged operative time is positively associated with post CS infection [20, 22]. Similar to our findings, the duration of labor prior to CS was longer in the case group compared to control [10, 20, 23]*.* Cases complicated by IAI in our study were more likely to have lost more blood intraoperatively, which suggests that bleeding may predispose to infectious morbidity; other authors made similar observations [10, 20, 24]*.* Furthermore, we found that chorioamnionitis and PROM are associated significantly with high incidence of IAI; these findings were confirmed by many authors [10, 19, 20, 25]*.*

From the obtained results, the mortality rate in the IAI case group was 1.22% due to septicemia. It has been reported that post cesarean infections may increase maternal morbidity and mortality [26, 27]. In addition, septicemia is the 3rd most common cause of maternal mortality (10.7%) [28]. The rate obtained in our study was lower than that obtained by Acosta et al., who reported a rate of 4.6% after cesarean delivery in a national cohort study [29]. Additionally, this rate is lower than that reported in some developing countries, such as Benin (27.4%) and Nigeria (16%) [30, 31].

The present results revealed that prolonged CS operative time (> 1 h) was associated with 3.4 times increased odds of IAI. Other studies reported the same trend. Wodajo et al. found that prolonged duration of CS (> 1 h) is significant risk factor for SSI (AOR = 12.32, CL (5.46–27.77) [32]. Additionally, in Tanzania, long operative time was significantly associated with the outcome with a hazard ratio of 2.3 [15]. It has been reported that prolonged CS surgery duration may increase the risk of exogenous contamination, which could lead to increased infection [33].

The study also showed a significant correlation between blood loss (> 1000 ml) and IAI (AOR 2.86; 95% CI =2.04–3.92; *p* = 0.011). Generally, excessive blood loss reduces immunity and leads to a lowering of hemoglobin concentration, which increases the risk of infection by negatively affecting macrophage activity [33] and impeding wound healing progress [34]. Similar results were shown from studies from Nigeria [35], India [36] and China [37].

In our study, prolonged labor (greater than 24 h.) was noted to be an independent risk factor for post cesarean IAI. This result is further supported by a study in Ethiopia by Gelaw et al., who studied SSI and its associated risk factors following cesarean section and found that duration of labor was one of the identified independent risk factors for surgical site infections, with an AOR of 3.48 [38]. Additionally, Krieger et al. and Moulton et al. found similar results [20, 25]. Prolonged labor increased the number of vaginal examinations, which consequently increases the chance of iatrogenic contamination during examination [39].

Chorioamnionitis was found to be the most significant risk factor for IAI in the present study, (AOR = 9.54; 95% CI =6.15–16.2; *p* ≤ 0.001). Similar results were found in a recent study by Dotters-Katz et al., who studied the risk factors for post CS wound infection in the setting of chorioamnionitis. They found that 15.0% of women with clinical chorioamnionitis developed infections [40]. Kawakita and Landy reported similar findings [6].

The present study illustrated that PROM is the second most significant identifiable risk factors for post cesarean section IAI. Women with PROM prior to cesarean section were 7.5 times more likely to have IAI than controls (AOR = 7.54; 95% Cl (5.69, 10.24). These findings are in line with many studies in both developing and developed countries [6, 9, 25]*.* The possible explanation of this is that when the membranes rupture, the amniotic fluid, which is not sterile, may act as a transport medium by which pathogens may come into contact with the uterine and skin incisions which cause infection [41]. Furthermore, it has been well documented that both prolonged labor and rupture of membranes contribute to amniotic fluid colonization by the normal flora of the lower genital tract, leading to wound and peritoneal cavity contamination [32]. In contrast, Al Jama found that PROM did not reach a significant effect to be a risk factor for SSI [42]. Generally, previous studies confirmed our results by identifying a number of risk factors associated with increased rate of post CS infection, such as: DM, chorioamnionitis, PROM, emergency delivery, longer operative time and lack of antenatal care [21, 43–45]*.* Finally, active efforts must be undertaken by healthcare institutions to implement measures aimed at decreasing the risk of infection. Some reports suggest that implementing quality improvement measures led to a significant reduction in post CS infection at an institutional level [46, 47].

Retained (‘missed’) towels are a preventable problem. Several measures in our institution are in place to avoid this problem, e.g., a high index of suspicion, a multidisciplinary approach during surgical procedures, using radio-opaque swabs, wound exploration, counting the swabs at the start and before the end of the surgery and documenting this information in the notes and applying risk management policies.

To the best of our knowledge, this is the first study done in our locality about IAI following CS with large number of participants (cases and controls) in a tertiary referral hospital serving a large population and over a long period. These are regarded the main strengths of this study.

This study has some limitations, including factors related to the retrospective design. For example, the present results are restricted only to cases of IAI that were diagnosed within the studied hospital, meaning that we could not survey cases who were diagnosed and treated outside, which may falsely lower the overall obtained rate of IAI. Another limitation is that we could not include some risk factors for IAI such as nutrition status. Additionally, we did not include data about the organisms that caused IAI or data about antibiotic prophylaxis or treatment or type of antimicrobial skin preparation. An additional limitation is that generalizability may be limited because data were collected from one hospital, although the studied hospital is the main tertiary referral hospital in our region. Despite these limitations, we feel that our findings are very important and will be applicable within a wider population.

## Conclusions

In conclusion, the results of our study revealed that the overall incidence of IAI post CS was 2.87%, and the mortality rate was 1.2%. The recorded risk factors and predictors for IAI in our study were chorioamnionitis, PROM, duration of CS (> 1 h), no ANC visits, emergency CS, blood loss (> 1000 ml), prolonged duration of labor (≥24 h.) and diabetes mellitus. Intra-abdominal infection post CS is a major problem in developing countries, so great effort must be taken to reduce this burden. Future prospective studies are warranted focusing on this issue.

## Data Availability

The datasets used and/or analyzed during the current study are available from the corresponding author on reasonable request.

## Abbreviations

ANC: Antenatal care
AOR: Adjusted odds ratio
BMI: Body mass index
CI: Confidence interval
COR: Crude odds ratio
CS: Cesarean section
DM: Diabetes Mellitus
IAI: Intra-abdominal infection
PROM: Premature rupture of membranes
SD: Standard deviation
SSI: Surgical site infection
